# Effects of Cigarette Smoke on the Nasal Respiratory and Olfactory Mucosa in Allergic Rhinitis Mice

**DOI:** 10.3389/fnins.2020.00126

**Published:** 2020-02-18

**Authors:** Rumi Ueha, Satoshi Ueha, Kenji Kondo, Hironobu Nishijima, Tatsuya Yamasoba

**Affiliations:** ^1^Department of Otolaryngology, The University of Tokyo, Tokyo, Japan; ^2^Division of Molecular Regulation of Inflammatory and Immune Diseases, Research Institute for Biomedical Sciences, Tokyo University of Science, Chiba, Japan

**Keywords:** cigarette smoke, allergic rhinitis, respiratory mucosa, olfactory epithelium, ovalbumin

## Abstract

**Objective:**

Cigarette smoke (CS) exposure reportedly enhances allergic airway inflammation. However, some studies have shown an association between current cigarette smoke exposure and a low risk for allergic rhinitis. Thus, the impact of CS exposure on allergic rhinitis remains poorly understood. The purpose of this study was to investigate the effects of CS on the respiratory mucosa (RM) and the olfactory epithelium (OE) of mice with allergic rhinitis, as the effects may differ depending on the nasal histological compartments.

**Methods:**

Eight-week-old male BALB/c mice were used for this study. We developed a mouse model of smoking by intranasally administering 10 doses of a CS solution (CSS), and a mouse model of allergic rhinitis by sensitization with intraperitoneal ovalbumin (OVA) injection and intranasal challenge with OVA. We examined the effects of CS on the nasal RM and OE in mice with or without allergic rhinitis using histological, serum, and genetic analyses. First, we examine whether CSS exposure induces allergic responses and then, examined allergic responses in the OVA-sensitized allergic rhinitis mice with or without CSS exposure.

**Results:**

Short-term CSS administration intensified allergic responses including increased infiltration of eosinophils and inflammatory cells and upregulation of interleukin-5 expression in the nasal RM of OVA-immunized mice, although only CSS induced neither allergic responses nor impairment of the RM and OE. Notably, repetitive OVA-immunization partially impaired the OE in the upper-lateral area, but CSS administration did not reinforce this impairment in OVA-induced allergic mice.

**Conclusion:**

Short-term CSS exposure strengthened allergic responses in the nasal RM and did not change the structure of the OE. These results suggest that patients with allergic rhinitis could experience exacerbation of allergic symptoms after CS exposure.

## Introduction

Allergy is a predisposing factor for multiple respiratory conditions including allergic rhinitis and asthma (Gelber et al., 1993; Monico et al., 2019). Cigarette smoke (CS) is a risk factor of developing allergic diseases, mediated by excessive immune responses to various allergens, and impacts the immune system (Mehta et al., 2008). It is well known that components of CS, such as nicotine, tar, carbon dioxide, formaldehyde, and acrolein, exacerbate pathogenic immune responses or disrupt innate and adaptive immunity (Mehta et al., 2008; Qiu et al., 2017).

Some studies have shown an association between current CS exposure and a low risk for allergic rhinitis (Hjern et al., 2001; Nagata et al., 2008) and that the prevalence of allergic asthma and allergic rhinitis decreased with increased exposure to CS (Hjern et al., 2001). Conversely, CS reportedly increases nasal allergic responses with concomitant increase in the serum immunoglobin E (IgE) level and production of interleukin-5 (IL-5) (Oryszczyn et al., 2000; Saulyte et al., 2014; Kim et al., 2017). In addition, CS directly affects the epithelial cells and results in increased permeability, mucus overproduction, increased release of proinflammatory cytokines and chemokines, enhanced recruitment of neutrophils, and disturbed lymphocyte balance toward T helper type 2 (Th2) cells (Strzelak et al., 2018). Recently, smoking has been recognized as a cause of acute eosinophilic pneumonia (AEP), which can occur within a few weeks or months of initiating smoking or even as a result of passive smoking exposure (De Giacomi et al., 2017). This may imply that smoking can amplify allergic responses, as eosinophilia is strongly associated with allergy. The effects of smoking on allergic rhinitis have been mainly discussed and verified on the serum level and in *in vitro* studies, and the influence of cigarette-smoke exposure on allergic rhinitis is not entirely understood; *in vivo* studies are indispensable to elucidate the effects of smoking on allergic responses in the nasal mucosa.

The nasal mucosa is divided into the respiratory mucosa (RM) and the olfactory mucosa based on its histological components and functions. The RM consists of various types of epithelial cells including ciliated columnar and goblet cells, and is mainly affected by allergic inflammation and viral infections (Wang et al., 2008). The olfactory mucosa serves olfaction and consists of the olfactory epithelium (OE) and the sub-epithelium tissue. The number of mature olfactory receptor neurons (ORNs) in the OE is closely related to the degree of olfaction (Ueha et al., 2018a), and the ORNs are classified into four groups according to their zonal expression patterns (Mori et al., 2000). Regarding the impact of smoking on the OE, we previously reported that CS impaired the OE and olfaction by reducing the olfactory progenitor cells and mature ORNs (Ueha et al., 2016a) and that CS delayed regeneration of the OE (Ueha et al., 2016b). However, the effects of CS on each area of the allergic nasal mucosa have not been verified.

In the present study, we explored the effects of CS on the allergic RM and OE using mouse models of smoking that involved intranasal administration of a CS solution (CSS) and allergic rhinitis immunization by ovalbumin (OVA).

## Materials and Methods

### Mice

Eight-week-old male BALB/c mice were purchased from Saitama Experimental Animals (Saitama, Japan). Mice were housed in a temperature-controlled environment under a 12-h light–dark cycle with *ad libitum* access to food and water. All experiments were conducted in accordance with institutional guidelines and with the approval of the Animal Care and Use Committee of the University of Tokyo (No. P15-113).

### Mouse Model of Allergic Rhinitis and Smoking

Fifty-five mice were used for this study. Twenty two mice were divided for comparison in allergic responses between the control and CSS exposure groups. The remaining 33 mice were allocated into three groups; control, OVA, OVA + CSS (*n* = 11, each group).

**FIGURE 1:**
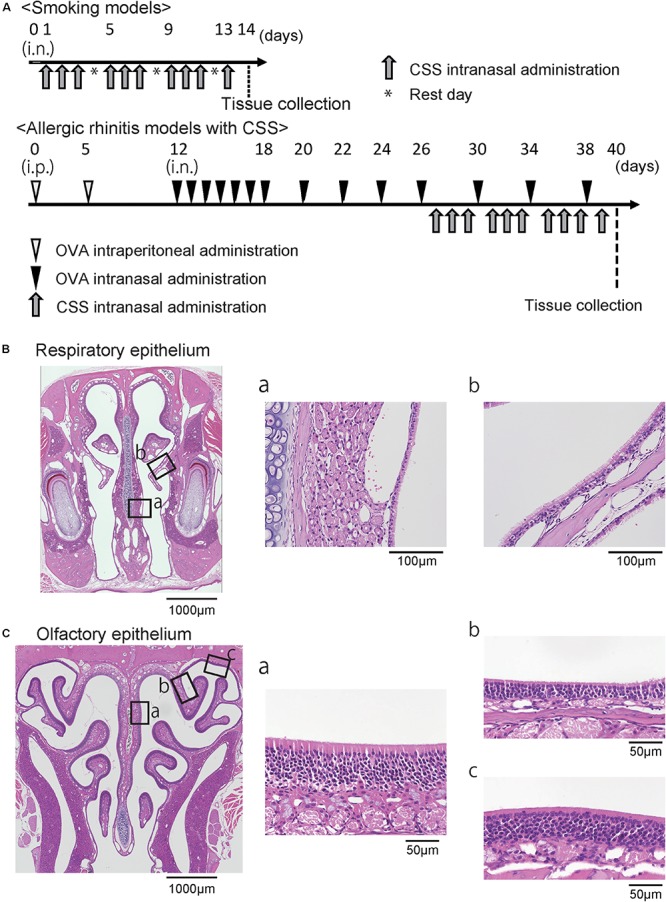
**(A)** Experimental timeline. **(B)** Representative images of hematoxylin and eosin stained sections of the respiratory epithelium from control mice (40× magnification). The boxes in **(B)** indicate the region of the respiratory epithelium shown at higher magnification (400× magnification) in **(B-a,b)**. **(C)** Representative images of the olfactory epithelium. The boxes in **(C)** indicate the region of the olfactory epithelium shown at higher magnification in **(C-a,b,c)**. OVA, ovalbumin; CSS, cigarette smoke solution; i.p., intraperitoneal.

Airway allergy was induced as previously described with some modification (Kim et al., 2011; Nakanishi et al., 2013). In brief, OVA (grade V; Sigma, St. Louis, MI, United States) was used to sensitize and challenge the mice. Mice were immunized with intraperitoneal injection of 100-μg OVA in 4-mg aluminum hydroxide gel (Imject Alum; Pierce, Rockford, IL, United States) on days 0 and 5, followed by a daily intranasal challenge with OVA diluted in sterile normal saline (100 μg OVA/10 μL per mouse) from days 12 to 18. Thereafter, prolonged continuous inflammation was maintained by subsequent nasal exposure of 10-μL OVA dilution (100 μg) every 4 days until day 38 (Figure 1A). The control mice received saline instead of OVA.

The CSS was prepared as previously reported (Ueha et al., 2016a). The CSS was produced by bubbling a stream of the smoke of Hi-Lite cigarettes (tar yield: 17 mg/cigarette, nicotine yield: 1.4 mg/cigarette; Japan Tobacco Inc., Tokyo, Japan) through saline (1 mL/cigarette) purchased from Cmic Bioresearch Center Co., Ltd (Yamanashi, Japan). The CSS was administered intranasally (20 μL/animal/time) once a day on days 27 to 29, 31 to 33, 35 to 37, and 39, i.e., 10 doses in total (Figure 1A). Control mice received saline intranasally according to the same schedule used for the CSS mice. The mice were sacrificed under general anesthesia by intraperitoneal ketamine-xylazine injection on day 40.

### Tissue Preparation

The nasal specimens from the incisors to the olfactory bulb and the septal nasal mucosa were harvested on day 40 for histological and quantitative polymerase chain reaction (qPCR) analyses. Immediately after sacrificing the mice, the nasal cavities were gently irrigated with 4% paraformaldehyde in order to minimize mechanical damage to the OE. After decalcification, the tissues were dehydrated in a series of graded ethanol solutions and then embedded in paraffin.

### Antibody Staining

Anti-mouse primary antibodies were prepared for tissue staining as follows: CD3 (1:300 dilution; rabbit monoclonal (SP7), Nichirei Corporation #413601; Nichirei Corporation, Tokyo, Japan), Ki67 (1:200 dilution; rabbit monoclonal, Novus #NB600-1252; Novus, Littleton, CO, United States), neutrophil (1:300 dilution; rat monoclonal, Abcam 2557; Abcam, Tokyo, Japan), and olfactory marker protein (OMP; 1:8000 dilution, goat polyclonal, Wako, Tokyo, Japan). CD3 is a complex of polypeptides that form part of the T cell receptor and a marker for T cells in tissue sections, as CD3 is expressed at all stages of T-cell development (Clevers et al., 1988). The Ki67 protein is a cellular marker of proliferation strictly associated with cell proliferation (Duchrow et al., 1995), and Ki67-positive cells are detected throughout the depth of the nasal mucosa. Especially in the OE, such cells are observed mainly in the basal layer (Ueha et al., 2016a, 2018b). OMP is exclusively expressed in mature ORNs (Buiakova et al., 1996).

### Histological Analyses

As previously described (Ueha et al., 2014, 2016a, 2018b; Nishijima et al., 2016), all samples were cut at the level of the incisive papilla of the hard palate in coronal sections for examinations of the RM (Nishijima et al., 2016), and were cut from the level of the anterior end of the olfactory bulb to the 1-mm anterior level for examinations of the OE (Ueha et al., 2014, 2016a, 2018b). Four-micrometer thick paraffin sections were deparaffinized in xylene and rehydrated in ethanol before hematoxylin and eosin staining for evaluation of whole tissue structure, Sirius red staining for eosinophils, periodic acid-Schiff and Alcian blue (PAS/AB) staining for goblet cells, and immunostaining. Prior to immunostaining, deparaffinized sections were treated with 3% hydrogen peroxide to block endogenous peroxidase activity and were incubated in Blocking One (Nacalai Tesque, Kyoto, Japan) to block non-specific immunoglobulin binding. Primary antibodies were detected using peroxidase-conjugated secondary antibodies and a diaminobenzidine substrate. The RM and three different coronal sections of the OE located at 500-μm intervals were captured using a digital microscope camera (Keyence, Osaka, Japan, BZ-9000) with a 40 × objective lens (Figure 1B).

Regarding the RM, the number of eosinophils, CD3^+^ cells, neutrophils, and Ki67^+^ cells per high-power microscopic field (10 × 40) were counted from two sampling areas (nasal septum and lateral nasal concha) for each section using the hybrid cell count system (Keyence) in a blinded manner (Figure 1B). The number of goblet cells per 500 μm of the respiratory epithelium was counted, and the heights of the columnar respiratory epithelial cells (CRECs) were measured from the basal part to the superficial part of the CREC by averaging the five points at every 100 μm of the respiratory epithelium. Analyses for the OE were restricted to the OE of the nasal septum (NS), the upper lateral area (UL), and the uppermost lateral area (UML) to minimize variation among specimens (Figure 1C). The number of OMP^+^ ORNs per 500 μm of NS or 300 μm of UL in the OE was quantified by averaging the number of cells on the right and left sides of each three sections in a blinded manner.

### Enzyme-Linked Immunosorbent Assay (ELISA)

Sera from mice were prepared and 500-fold serum dilutions were tested for total IgE and OVA-specific IgE to confirm the presence of allergic responses using the LBIS Mouse IgE ELISA Kit (# 633-02899, Fujifilm, Gunma, Japan) and LBIS Mouse anti-OVA-IgE ELISA Kit (# 633-07659, Fujifilm, Gunma, Japan), according to the manufacturer’s instructions.

### Quantitative Real-Time Polymerase Chain Reaction

Total RNA was isolated from the septal nasal mucosa using TRIzol reagent (Life Technologies, Gaithersburg, MD, United States) on day 40 and then converted to cDNA using the ReverTra Ace qPCR RT Master Mix with gDNA Remover (Toyobo, Osaka, Japan) according to the manufacturer’s instructions. qPCR analysis was performed using the THUNDERBIRD Probe qPCR Mix or THUNDERBIRD SYBR qPCR Mix (Toyobo) and an ABI 7500 sequence detector system (Life Technologies). The gene-specific primers and probes used were: *Rps3* as endogenous control (Life Technologies assay number Mm00656272_m1), interleukin 1 beta (*Il1b)* (forward 5′-AAATACCTGTGGCCTTGGGC-3′, reverse 5′-TCT TCTTTGGGTATTGCTTGGGA-3′), tumor necrosis factor-alfa (*Tnfa)* (forward 5′-GTAGCCCACGTCGTAGCAAA-3′, reverse 5′-TTGAGATCCATGCCGTTGGC-3′), *Il4* (forward 5′-TCGGC ATTTTGAACGAGGTC-3′, reverse 5′-CTGTGGTGTTCTTCG TTGCTG-3′), *Il5* (forward 5′-TTGACCGCCAAAAAGAGAA GTG-3′, reverse 5′-CTCAGCCTCAGCCTTCCATT-3′), *Il13* (forward 5′-AGCATGGTATGGAGTGTGGAC-3′, reverse 5′-GC TGGAGACCGTAGTGGGG-3′), *Il17* (forward 5′-GGACTCTC CACCGCAATGAA-3′, reverse 5′-TTTCCCTCCGCATTGACA CA-3′), *Il25* (forward 5′-TGGAGCTCTGCATCTGTGTC-3′, reverse 5′-GATTCAAGTCCCTGTCCAACTC-3′), and *Il33* (forward 5′-GGTGAACATGAGTCCCATCA-3′, reverse 5′- CGTCACCCCTTTGAAGCTC -3′). The expression levels of each gene were normalized to the level of *Rps3* expression for each sample.

### Statistical Analysis

Statistical analyses were performed using the Mann–Whitney *U* test for comparisons between groups or with one-way analysis of variance with *post hoc* Tukey’s tests for comparisons among multiple groups using GraphPad Prism software (version 6.0; GraphPad Software, Inc., San Diego, CA, United States)^1^. qPCR data were subjected to logarithmic transformation prior to analysis. *P* < 0.05 was considered to be statistically significant.

## Results

### Short-Term CSS Administration Induces Neither Allergic Responses nor Impairment in the RM and OE

**FIGURE 2:**
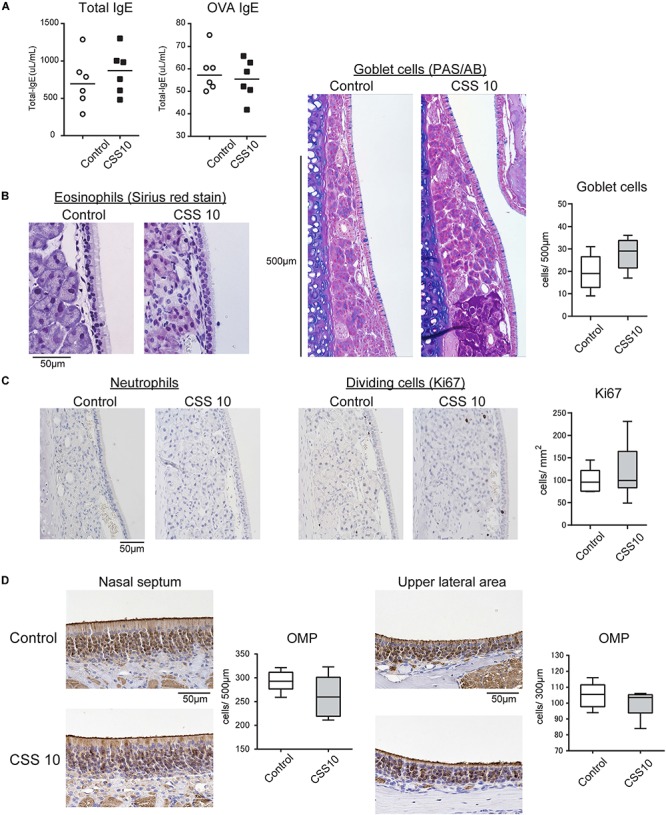
**(A)** Serum immunoglobin E (IgE) levels of the control and cigarette smoke solution (CSS)-treated mice were determined by enzyme linked immunosorbent assay. **(B)** Representative images of Sirius red staining for eosinophils and periodic acid-Schiff and Alcian blue (PAS/AB) staining for goblet cells in the nasal RM of the control mice and mice treated with 10 doses of CSS (CSS 10). **(C)** Representative immunohistochemical images of neutrophils and Ki67^+^ dividing cells in the nasal RM (400× magnification), and comparative charts of Ki67^+^ cell counts (*n* = 6, Mann–Whitney *U* test). **(D)** Representative images of olfactory marker protein (OMP)^+^ mature olfactory receptor neurons (ORNs) in two different areas of the olfactory epithelium: the nasal septum and upper lateral area. There were no significant differences in the number of OMP^+^ mature ORNs between the control and CSS-treated mice (*n* = 6, Mann–Whitney *U* test). OVA, ovalbumin.

To investigate the immunological and pathological effects of CSS treatment on allergic rhinitis, we began with short-term CSS treatment models to examine whether CSS treatment itself induces allergic responses and allergy-associated histological changes. We examined the effect of 10 doses (once a day, two doses for 3 days) of CSS intranasal administration on the nasal RM and OE. There were no significant changes in the serum levels of total IgE and OVA-specific IgE between the control and CSS 10 groups (Figure 2A). In the RM, neither eosinophil and neutrophil infiltration nor appreciable allergic-related tissue changes, such as changes in the number of goblet cells and the epithelial height, were recognized in either the control or CSS mice, although an increasing tendency in the number of the goblet cells with 10 doses of CSS administration was recognized (Figure 2B). There were no significant differences in the number of dividing cells between the groups (Figure 2C). In addition, we observed no significant change in the number of OMP^+^ mature ORNs in the OE (Figure 2D).

### CSS Exposure Enhances the OVA-Induced Allergic Responses in the Nasal RM

**FIGURE 3:**
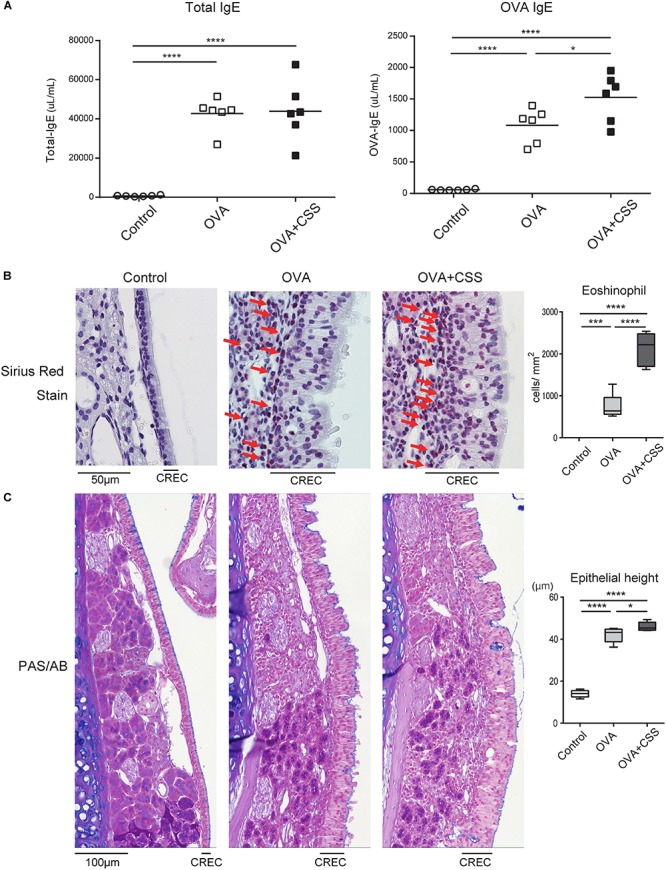
**(A)** Serum immunoglobin E (IgE) levels in the control, ovalbumin-induced allergic (OVA), and OVA-allergic-smoking [OVA + cigarette smoke solution (CSS)] groups were determined by enzyme linked immunosorbent assay. **(B)** Representative images of Sirius red staining for eosinophils in the nasal respiratory mucosa (RM) (red arrows) (400× magnification), and a comparative chart of the eosinophil counts in each group. **(C)** Representative images of periodic acid-Schiff and Alcian blue (PAS/AB) staining in the nasal RM (400× magnification), and a comparative chart of the height of the columnar respiratory epithelial cells (CRECs). The height of CRECs in the OVA-immunized group was higher than that in the control group, and that in the OVA + CSS group was highest among the groups. *P < 0.05; ***P < 0.001; ****P < 0.0001 (*n* = 6, one-way analysis of variance).

As CS may influence airway allergic responses, we next examined allergic responses in the OVA-sensitized allergic rhinitis mice with or without CSS exposure. The magnitude of the allergic response was evaluated by measuring IgE serum levels and counting the number of eosinophils infiltrating the nasal RE. The OVA-immunized group showed significantly higher IgE serum levels (total IgE and OVA-specific IgE) than those observed in the control group (*p* < 0.0001), suggesting the successful induction of an allergic response. Moreover, CSS induced elevation of serum OVA-specific IgE (*p* = 0.013), but not of serum total IgE (Figure 3A). Histologically, many eosinophils were seen to infiltrate the subepithelial tissue in the OVA group, and the number of infiltrating eosinophils was larger in the OVA with CSS exposure (OVA + CSS) group than in the OVA group (*p* < 0.0001) (Figure 3B). OVA sensitization and challenge induced an increase in the height of the ciliated columnar respiratory epithelial cells (*p* < 0.0001), and CSS exposure reinforced this response (*p* = 0.036), suggesting that CSS might boost OVA-induced allergic responses. However, goblet cells were not visible in the respiratory epithelium either in the CSS mice or in the OVA + CSS mice (Figure 3C).

### CSS Increases Inflammatory Cell Infiltration and Cell Division With the Upregulation of Il1b and Il5 in the Nasal RM of OVA-Induced Allergic Mice

**FIGURE 4:**
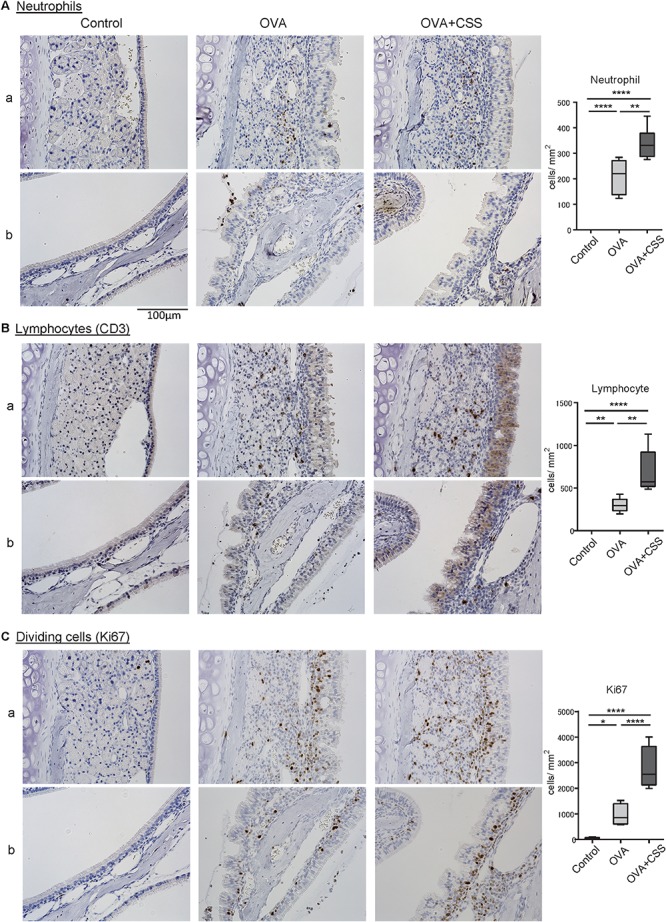
**(A–C)** Inflammatory cell infiltration and cell division in the nasal RM were evaluated using immunohistochemical staining (brown). Tissue sections were counterstained with the nuclear dye hematoxylin (blue). Representative immunohistochemical images of neutrophils **(A)**, CD3^+^ lymphocytes **(B)**, and Ki67^+^ dividing cells **(C)** (400× magnification), and comparative charts of cell counts in each group. *P < 0.01; **P < 0.01; ****P < 0.0001 (*n* = 6, one-way analysis of variance). OVA, ovalbumin; CSS, cigarette smoke solution.

**FIGURE 5:**
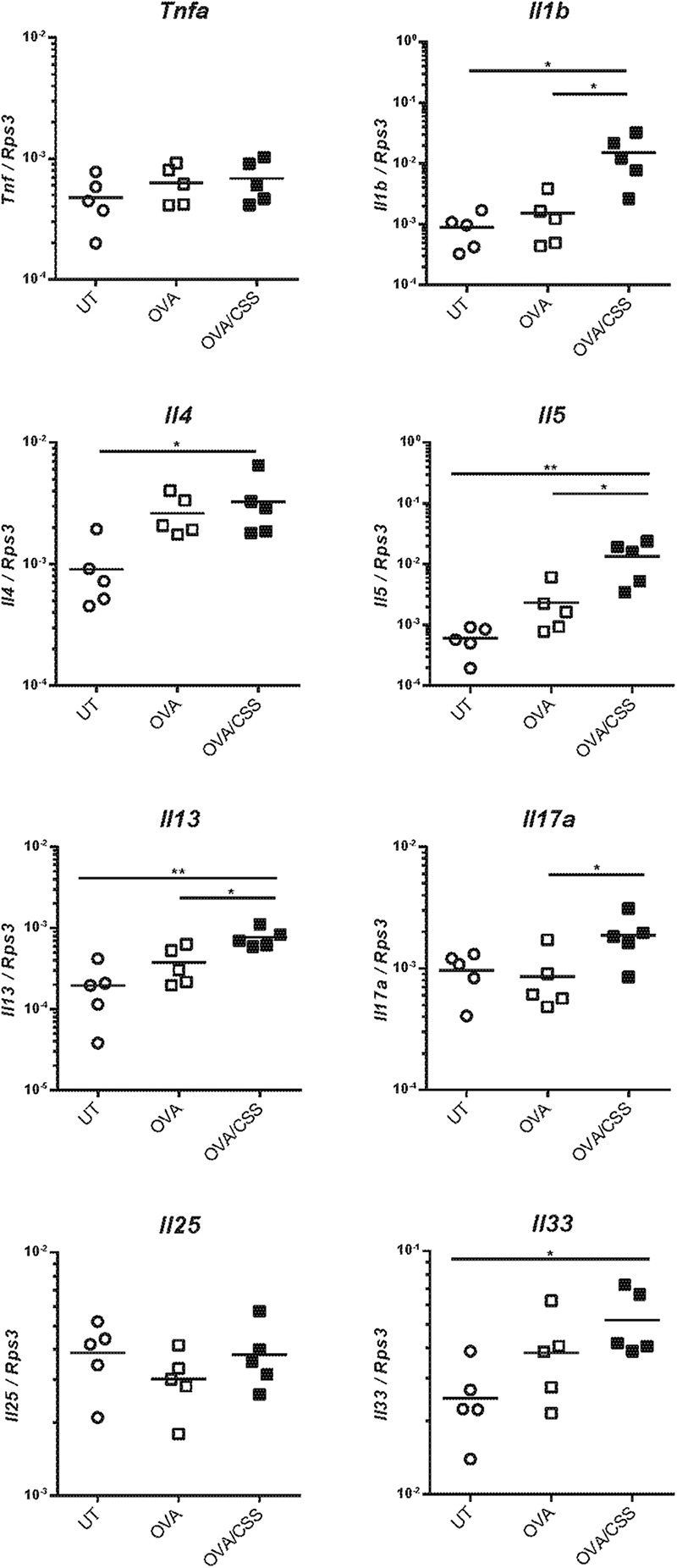
*Tnfa*, *Il1b*, *Il4*, *Il5*, *Il13*, *Il17*, *Il25*, and *Il33* expression levels in the nasal mucosa were quantified by real-time quantitative reverse transcription polymerase chain reaction and are expressed relative to the expression of the endogenous control gene *Rps3* (*n* = 5; *P < 0.05, **P < 0.01, one-way analysis of variance). Tnfa, tumor necrosis factor-alfa; Il1b, interleukin-1β; Il4, interleukin-4; Il5, interleukin-5; Il13, interleukin-13; Il17, interleukin-17; Il25, interleukin-25; Il33, interleukin-33; OVA, ovalbumin; CSS, cigarette smoke solution.

Next, to elucidate the molecular backgrounds of inflammatory responses in the OVA-immunized mice and the impact of CSS administration on the allergic responses, we investigated inflammatory cell infiltration, specifically of neutrophils and lymphocytes, and cell division in the nasal RM. In the OVA group, neutrophils and CD3^+^ lymphocytes infiltrated the epithelial and subepithelial tissue of the RM, although no neutrophils and CD3^+^ lymphocytes infiltrated the RM of the control mice. Moreover, the numbers of neutrophils and CD3^+^ lymphocytes infiltrating in the OVA + CSS group were significantly higher than those infiltrating the OVA alone group (*p* = 0.0019, 0.0013) (Figures 4A,B). Ki67 + dividing cells were rarely detected in the RM of control mice, whereas a large number of such cells were recognized in the RM of OVA-immunized mice, and this increase in number was more significant in the OVA + CSS mice (*p* < 0.0001) (Figure 4C). These results suggested that OVA induced inflammatory cell infiltration in the RM, which resulted in increase of cell division in the tissue. Then, to study the inflammatory mediators that contribute to nasal inflammation, we examined the expression levels of cytokines that are involved in inflammation (*Tnfa*, *Il1b*) and in allergic responses (*Il4*, *Il5*, *Il13, Il17, Il25, Il33*) in the nasal mucosa. The *Il1b*, *Il5*, *Il3*, and *Il17* expression levels were higher in the OVA + CSS group than in the OVA group (*p* = 0.021, 0.014, 0.015, 0.045, respectively), and the *Il4* and *Il33* expression levels were higher in the OVA + CSS group than in the untreated group (*p* = 0.035, 0.025, respectively). However, there were no significant differences in the expression levels of *Il25* among groups (Figure 5). These results suggested that CSS administration exacerbated allergic inflammation by enhancing the production of inflammatory and allergic cytokines.

### Repetitive OVA-Immunization Partially Impairs OMP^+^ Mature ORNs in the OE at the Upper-Lateral Area

**FIGURE 6:**
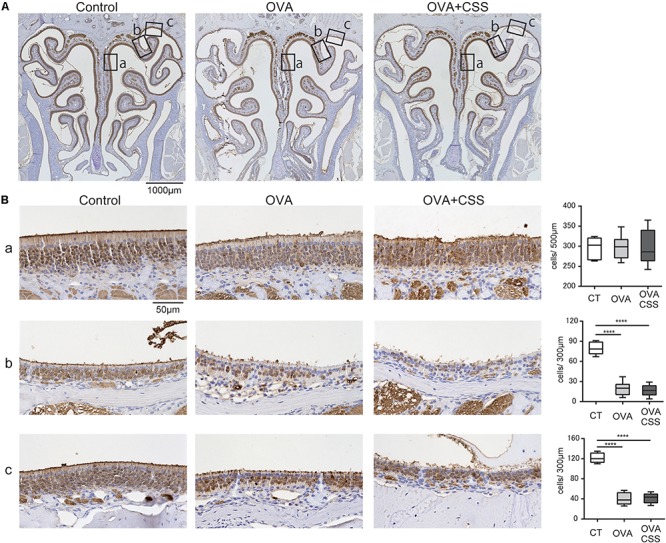
**(A,B)** Representative immunohistochemical images of olfactory marker protein (OMP)^+^ mature olfactory receptor neurons (ORNs) in the olfactory epithelium **(A)** 40× magnification; **(B)** 400× magnification). Each box **(a–c)** in **(A)** indicates the region of the olfactory epithelium shown at a representative higher magnification in **(B,a)** nasal septum; **(b)** upper-lateral area; **(c)** uppermost-lateral area. Comparative charts of cell counts of OMP^+^ ORNs in each group are shown. ****P < 0.0001 (*n* = 6, one-way analysis of variance). OVA, ovalbumin; CSS, cigarette smoke solution.

Before investigating the CSS effects on the OE in allergic mice, we preliminary verified whether repetitive OVA-immunization damaged the OE. Although we previously reported that a cockroach allergen did not affect the number of OMP^+^ mature ORNs (Ueha et al., 2014), the examined area was restricted to the OE of the nasal septum. As the ORNs in the OE differ according to their zonal expression patterns, in the present study, we investigated the number of ORNs in the OE of three different areas; nasal septum (NS), named “upper-lateral area” (UL), and “uppermost-lateral area” (UML) (Figure 6A-a). There was no difference in the number of the OMP^+^ mature ORNs in the NS between the control and OVA groups (Figure 6B-a); however, OMP^+^ ORN numbers were significantly decreased in the UL and UML of OVA-immunized allergic mice (Figures 6B-b,c). These data suggest that OVA-induced allergic responses partially damage the OE, possible focally in the UL and UML.

### Short-Term CSS Administration Does Not Reinforce Partial OE Damage in OVA-Induced Allergic Mice

Finally, we examined whether short-term CSS exposure impaired the ORNs of the OE in the allergic rhinitis mice. No significant differences were found between the OVA and OVA + CSS groups in the number of the OMP^+^ mature ORNs (Figure 6B), suggesting that short-term CSS administration does not have a negative impact on the OE of the OVA allergic mice.

## Discussion

In the present study, we demonstrated that short-term CSS exposure (10 doses) did not cause nasal-tissue changes in the non-OVA-immunized mice; however, there were intensified allergic responses including increased eosinophil infiltration, elongation of the epithelial height, and upregulation of *Il5* and *Il13* expression, in the nasal RM of the OVA-immunized mice. In addition, 10 doses of CSS did not have any effects on OMP^+^ ORNs in the OE of allergic mice, although only allergic mice induced by OVA frequent attacks showed significant decrease in the number of OMP^+^ ORNs in the UL and UML OE areas, but not in the NS. These results suggest that the impact of CSS exposure on the allergic nasal mucosa may differ by histological compartment.

The respiratory mucosa exists both in the nose and in the trachea and bronchi (Brand-Saberi and Schafer, 2014). Recently, AEP, which is most typically caused by cigarette smoking, has been reported and gradually became known (Allen et al., 1989; Shiota et al., 2000; De Giacomi et al., 2017). CS exposure is recognized as a cause of AEP, which can occur within a few weeks or months of starting smoking, smoking resumption after quitting, and passive smoking (De Giacomi et al., 2017). Pulmonary eosinophilic inflammation can suddenly occur after CS exposure, and then patients with AEP can progress from mild dyspnea to life-threatening respiratory failure (Watanabe et al., 2002; De Giacomi et al., 2017). Histologically, marked eosinophil infiltration in the respiratory tissue is common. The pathogenesis of AEP reportedly starts with antigenic stimulation of T-lymphocytes and, more specifically, Th2 lymphocytes (Nureki et al., 2005). AEP can be rapidly cured by corticosteroid administration, and patients will clinically improve within 2–48 h of the first dose of corticosteroids. As there is the respiratory tissue in the nose, this type of condition and eosinophilic inflammation could occur in the nose.

Cigarette smoke has been reported to induce inflammatory cell infiltration, especially of neutrophils, as well as cell division and mucus hypersecretion (Mizutani et al., 2009; Singh et al., 2011). However, the CSS doses used in the present study did not cause inflammatory cell infiltration or cell division, although an increasing tendency in the number of goblet cells could be recognized. Considering our previous study finding that the reduction in the number of olfactory mature cells was caused by 20 doses of CSS but not by 10 doses of CSS (Ueha et al., 2016a), 10 doses of CSS might be insufficient to induce histological inflammatory responses in the RM. An increase in leukocytes, such as eosinophils, neutrophils, and lymphocytes is usually identified in the RM of allergic rhinitis (Kim et al., 2011), and also in the present study, marked increases in inflammatory cells and cell division were observed in the OVA-immunized allergic mice. Additional 10 doses of CSS on allergic mice exaggerated inflammatory cell infiltration of CD3^+^ lymphocytes, neutrophils, and eosinophils, suggesting that CSS exposure in the allergic condition could induce appreciable histological changes, although the dose and number of CSS administrations were insufficient to influence non-allergic mice. Moreover, goblet cells could not be observed in either the OVA or OVA + CSS groups. This indicates that frequent OVA sensitization and challenge can induce hypertrophy of the ciliated columnar respiratory epithelial cells, but not mucus production.

**FIGURE 7:**
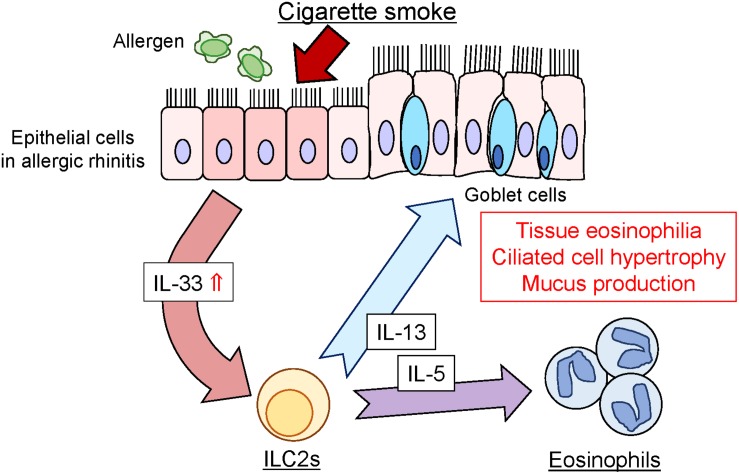
Schematic representation of CSS-induced enhancement of allergic responses in the nasal RM. CSS, cigarette smoke solution; ILC2, innate lymphoid cells; IL-5, interleukin-5; IL-13, interleukin-13; IL-33, interleukin-33.

Regarding the underlying mechanisms of CSS-induced enhancement of allergic inflammation, increase in IL-33 appears to be upstream of the CSS-induced allergic response. IL-33 is a member of the IL-1 cytokine family and is upregulated by cigarette smoke (Kearley et al., 2015). Upon epithelial or endothelial injury, IL-33 is released from damaged cells and induces type-2 innate immunity via activation of group 2 innate lymphoid cells (ILC2s) through its receptor ST2. Activated ILC2s produce Th2 cytokines, IL-5, and IL-13, in a synergistic manner (Chan et al., 2019). IL-5 and IL-13 have been reported to stimulate eosinophil activation and induce eosinophilia and ciliated epithelial cell hypertrophy in the respiratory tissue (Schmid-Grendelmeier et al., 2002; Takatsu and Nakajima, 2008; Tanabe et al., 2008). The results of the present study suggest that CSS administration could activate the production of inflammatory cytokines, particularly of IL-33, in OVA-induced allergic mice and enhance the production of IL-5 and IL-13. This type-2 cytokine cascade resulted in eosinophilic infiltration and ciliated epithelial cell hypertrophy in the nasal RM (Figure 7). Although CSS could exacerbate allergic responses via the above type-2 cytokine cascade in our experimental protocol, the generalizability of the findings should be investigated in other experimental settings with different timing, doses, and duration of CSS or OVA treatment. Considering that short-term CSS administration increased eosinophilic inflammation in allergic mice, rhinologic symptoms can be exacerbated in patients with allergic rhinitis by active and passive smoking. In such an eventually, systemic corticosteroid administration may be effective to reduce inflammation. Further research is needed to clarify which component of CS reinforces allergic responses.

Many previous reports have revealed that CS could augment allergic inflammation (Botelho et al., 2011; Saulyte et al., 2014; Strzelak et al., 2018); however, chronic smoking exerts a protective effect on allergic sensitization to some aeroallergens and prevents allergic diseases protecting against pollinosis (Hjern et al., 2001; Sopori, 2002; Nagata et al., 2008; Monico et al., 2019). Various components of CS suppress the Th2 cell response, which plays an important role in IgE production and in the development of immediate hypersensitivity (Ozaki et al., 2010), although the mechanism by which CS protects against allergic sensitization has not been well clarified. Thus, it is necessary to distinguish between “effects of CS on allergic inflammation” and “effects of chronic smoking on allergic sensitization”; the allergic symptoms of non-smoking patients with allergic rhinitis may worsen by exposure to CS.

Olfactory dysfunction in patients with allergic rhinitis is considered to be mainly caused by mechanical obstruction, but an inflammatory component has also been recently implicated (Wolfensberger and Hummel, 2002; Ozaki et al., 2010; Stuck and Hummel, 2015). Considering that eosinophilic chronic rhinosinusitis causes olfactory dysfunction (Ishitoya et al., 2010; Shah et al., 2016), it is reasonable to assume that damage to the OE may depend on the degree of the allergic response. Our observation that the number of mature ORNs decreased in the UL and UML areas of the OE of allergic mice supports this hypothesis. Moreover, various OVA challenge conditions, such as a dose, the number of administrations, and the administration period may affect impairment of the OMP^+^ mature ORNs in the UL area of the OE. In addition, more frequent or higher dose administration of CSS may cause extensive impairment of the OE even in allergic mice, as long-term CSS exposure damaged olfactory progenitors and decreased mature ORNs, resulting in deterioration of olfaction (Ueha et al., 2016a). Regarding tissue change in the OE of allergic rhinitis mice, as all previous studies focused on the OE of the NS, and not on the UL part of the OE (Ozaki et al., 2010; Carr et al., 2012; Ueha et al., 2014), the lateral area of the OE should be included in further investigations.

## Conclusion

We demonstrated that in allergic rhinitis mice, CSS exposure strengthened allergic responses in the nasal RM and did not cause additional structural change in the OE, excluding the upper lateral area of the OE. These findings provide a basis of experimental evidence underlying CS-induced exacerbation of allergic rhinitis and the possible negative effects of allergic responses on olfaction. The present study brings attention to possible exacerbation of allergic symptoms after CS exposure especially in non-smoking patients with allergic rhinitis.

## Data Availability Statement

The data that support the findings of this study are available from the corresponding author, upon reasonable request.

## Ethics Statement

The animal study was reviewed and approved by the Animal Care and Use Committee of the University of Tokyo (No. P15-113).

## Author Contributions

RU developed the concept, designed and performed the experiments, analyzed the data, produced the figures, and wrote the initial draft of the manuscript. SU, KK, and HN performed some of the experiments and analyzed the results. TY developed the concept, designed and critically revised the manuscript. All authors contributed to interpretation of the data and writing of the manuscript.

## Conflict of Interest

The authors declare that the research was conducted in the absence of any commercial or financial relationships that could be construed as a potential conflict of interest.

## Footnotes

1 www.graphpad.com

## References

[B1] Allen J. N. Pacht E. R. Gadek J. E. Davis W. B. (1989). Acute eosinophilic pneumonia as a reversible cause of noninfectious respiratory failure. *N. Engl. J. Med.* 321 569–574. 10.1056/nejm198908313210903 2761601

[B2] Botelho F. M. Llop-Guevara A. Trimble N. J. Nikota J. K. Bauer C. M. Lambert K. N. (2011). Cigarette smoke differentially affects eosinophilia and remodeling in a model of house dust mite asthma. *Am. J. Respir. Cell Mol. Biol.* 45 753–760. 10.1165/rcmb.2010-0404OC 21317378

[B3] Brand-Saberi B. E. M. Schafer T. (2014). Trachea: anatomy and physiology. *Thorac. Surg. Clin.* 24 1–5. 10.1016/j.thorsurg.2013.09.004 24295654

[B4] Buiakova O. I. Baker H. Scott J. W. Farbman A. Kream R. Grillo M. (1996). Olfactory marker protein (OMP) gene deletion causes altered physiological activity of olfactory sensory neurons. *Proc. Natl. Acad. Sci. U.S.A.* 93 9858–9863. 10.1073/pnas.93.18.9858 8790421 PMC38519

[B5] Carr V. M. Robinson A. M. Kern R. C. (2012). Tissue-specific effects of allergic rhinitis in mouse nasal epithelia. *Chem. Senses* 37 655–668. 10.1093/chemse/bjs048 22490702

[B6] Chan B. C. L. Lam C. W. K. Tam L. S. Wong C. K. (2019). IL33: roles in allergic inflammation and therapeutic perspectives. *Front. Immunol.* 10:364. 10.3389/fimmu.2019.00364 30886621 PMC6409346

[B7] Clevers H. Alarcon B. Wileman T. Terhorst C. (1988). The T cell receptor/CD3 complex: a dynamic protein ensemble. *Annu. Rev. Immunol.* 6 629–662. 10.1146/annurev.iy.06.040188.003213 3289580

[B8] De Giacomi F. Decker P. A. Vassallo R. Ryu J. H. (2017). Acute eosinophilic pneumonia: correlation of clinical characteristics with underlying cause. *Chest* 152 379–385. 10.1016/j.chest.2017.03.001 28286263

[B9] Duchrow M. Schluter C. Key G. Kubbutat M. H. Wohlenberg C. Flad H. D. (1995). Cell proliferation-associated nuclear antigen defined by antibody Ki-67: a new kind of cell cycle-maintaining proteins. *Arch. Immunol. Ther. Exp.* 43 117–121. 8744726

[B10] Gelber L. E. Seltzer L. H. Bouzoukis J. K. Pollart S. M. Chapman M. D. Platts-Mills T. A. (1993). Sensitization and exposure to indoor allergens as risk factors for asthma among patients presenting to hospital. *Am. Rev. Respir. Dis.* 147 573–578. 10.1164/ajrccm/147.3.573 8442589

[B11] Hjern A. Hedberg A. Haglund B. Rosen M. (2001). Does tobacco smoke prevent atopic disorders? A study of two generations of swedish residents. *Clin. Exp. Allergy* 31 908–914. 10.1046/j.1365-2222.2001.01096.x 11422156

[B12] Ishitoya J. Sakuma Y. Tsukuda M. (2010). Eosinophilic chronic rhinosinusitis in Japan. *Allergol. Int.* 59 239–245. 10.2332/allergolint.10-rai-0231 20657162

[B13] Kearley J. Silver J. S. Sanden C. Liu Z. Berlin A. A. White N. (2015). Cigarette smoke silences innate lymphoid cell function and facilitates an exacerbated type I interleukin-33-dependent response to infection. *Immunity* 42 566–579. 10.1016/j.immuni.2015.02.011 25786179

[B14] Kim D. W. Khalmuratova R. Hur D. G. Jeon S. Y. Kim S. W. Shin H. W. (2011). Staphylococcus aureus enterotoxin B contributes to induction of nasal polypoid lesions in an allergic rhinosinusitis murine model. *Am. J. Rhinol. Allergy* 25:e00255-61. 10.2500/ajra.2011.25.3727 22185735

[B15] Kim Y. S. Kim H. Y. Ahn H. S. Sohn T. S. Song J. Y. Lee Y. B. (2017). The association between tobacco smoke and serum immunoglobulin e levels in Korean adults. *Intern. Med.* 56 2571–2577. 10.2169/internalmedicine.8737-16 28883244 PMC5658521

[B16] Mehta H. Nazzal K. Sadikot R. T. (2008). Cigarette smoking and innate immunity. *Inflamm. Res.* 57 497–503. 10.1007/s00011-008-8078-6 19109742

[B17] Mizutani N. Fuchikami J. Takahashi M. Nabe T. Yoshino S. Kohno S. (2009). Pulmonary emphysema induced by cigarette smoke solution and lipopolysaccharide in guinea pigs. *Biol. Pharm. Bull.* 32 1559–1564. 10.1248/bpb.32.1559 19721232

[B18] Monico B. Gama J. M. R. Pastorinho M. R. Lourenco O. (2019). Tobacco smoke as a risk factor for allergic sensitization in adults: conclusions of a systematic review and meta-analysis. *J. Allergy Clin. Immunol.* 143 417–419. 10.1016/j.jaci.2018.07.040 30205187

[B19] Mori K. von Campenhause H. Yoshihara Y. (2000). Zonal organization of the mammalian main and accessory olfactory systems. *Philos. Trans. R. Soc. Lond. B Biol. Sci.* 355 1801–1812. 10.1098/rstb.2000.0736 11205342 PMC1692907

[B20] Nagata C. Nakamura K. Fujii K. Kawachi T. Takatsuka N. Oba S. (2008). Smoking and risk of cedar pollinosis in Japanese men and women. *Int. Arch. Allergy Immunol.* 147 117–124. 10.1159/000135698 18520156

[B21] Nakanishi W. Yamaguchi S. Matsuda A. Suzukawa M. Shibui A. Nambu A. (2013). IL-33, but not IL-25, is crucial for the development of house dust mite antigen-induced allergic rhinitis. *PLoS One* 8:e78099. 10.1371/journal.pone.0078099 24205109 PMC3808342

[B22] Nishijima H. Kondo K. Toma-Hirano M. Iwasaki S. Kikuta S. Fujimoto C. (2016). Denervation of nasal mucosa induced by posterior nasal neurectomy suppresses nasal secretion, not hypersensitivity, in an allergic rhinitis rat model. *Lab. Invest.* 96 981–993. 10.1038/labinvest.2016.72 27322954

[B23] Nureki S. Miyazaki E. Ando M. Kumamoto T. Tsuda T. (2005). CC chemokine receptor 4 ligand production by bronchoalveolar lavage fluid cells in cigarette-smoke-associated acute eosinophilic pneumonia. *Clin. Immunol.* 116 83–93. 10.1016/j.clim.2005.03.001 15925835

[B24] Oryszczyn M. P. Annesi-Maesano I. Charpin D. Paty E. Maccario J. Kauffmann F. (2000). Relationships of active and passive smoking to total IgE in adults of the epidemiological study of the genetics and environment of asthma, bronchial hyperresponsiveness, and atopy (EGEA). *Am. J. Respir. Crit. Care Med.* 161(4 Pt 1), 1241–1246. 10.1164/ajrccm.161.4.9905027 10764318

[B25] Ozaki S. Toida K. Suzuki M. Nakamura Y. Ohno N. Ohashi T. (2010). Impaired olfactory function in mice with allergic rhinitis. *Auris Nasus Larynx* 37 575–583. 10.1016/j.anl.2009.12.004 20346605

[B26] Qiu F. Liang C. L. Liu H. Zeng Y. Q. Hou S. Huang S. (2017). Impacts of cigarette smoking on immune responsiveness: up and down or upside down? *Oncotarget* 8 268–284. 10.18632/oncotarget.13613 27902485 PMC5352117

[B27] Saulyte J. Regueira C. Montes-Martinez A. Khudyakov P. Takkouche B. (2014). Active or passive exposure to tobacco smoking and allergic rhinitis, allergic dermatitis, and food allergy in adults and children: a systematic review and meta-analysis. *PLoS Med.* 11:e1001611. 10.1371/journal.pmed.1001611 24618794 PMC3949681

[B28] Schmid-Grendelmeier P. Altznauer F. Fischer B. Bizer C. Straumann A. Menz G. (2002). Eosinophils express functional IL-13 in eosinophilic inflammatory diseases. *J. Immunol.* 169 1021–1027. 10.4049/jimmunol.169.2.1021 12097410

[B29] Shah S. A. Ishinaga H. Takeuchi K. (2016). Pathogenesis of eosinophilic chronic rhinosinusitis. *J. Inflamm.* 13:11. 10.1186/s12950-016-0121-8 PMC4822241 27053925

[B30] Shiota Y. Kawai T. Matsumoto H. Hiyama J. Tokuda Y. Marukawa M. (2000). Acute eosinophilic pneumonia following cigarette smoking. *Intern. Med.* 39 830–833. 10.2169/internalmedicine.39.830 11030209

[B31] Singh S. P. Gundavarapu S. Pena-Philippides J. C. Rir-Sima-ah J. Mishra N. C. Wilder J. A. (2011). Prenatal secondhand cigarette smoke promotes Th2 polarization and impairs goblet cell differentiation and airway mucus formation. *J. Immunol.* 187 4542–4552. 10.4049/jimmunol.1101567 21930963 PMC3197944

[B32] Sopori M. (2002). Effects of cigarette smoke on the immune system. *Nat. Rev. Immunol.* 2 372–377. 10.1038/nri803 12033743

[B33] Strzelak A. Ratajczak A. Adamiec A. Feleszko W. (2018). Tobacco smoke induces and alters immune responses in the lung triggering inflammation, allergy, asthma and other lung diseases: a mechanistic review. *Int. J. Environ. Res. Public Health* 15:E1033. 10.3390/ijerph15051033 29883409 PMC5982072

[B34] Stuck B. A. Hummel T. (2015). Olfaction in allergic rhinitis: a systematic review. *J. Allergy Clin. Immunol.* 136 1460–1470. 10.1016/j.jaci.2015.08.003 26409662

[B35] Takatsu K. Nakajima H. (2008). IL-5 and eosinophilia. *Curr. Opin. Immunol.* 20 288–294. 10.1016/j.coi.2008.04.001 18511250

[B36] Tanabe T. Fujimoto K. Yasuo M. Tsushima K. Yoshida K. Ise H. (2008). Modulation of mucus production by interleukin-13 receptor alpha2 in the human airway epithelium. *Clin. Exp. Allergy* 38 122–134. 18028464 10.1111/j.1365-2222.2007.02871.x

[B37] Ueha R. Mukherjee S. Ueha S. de Almeida Nagata D. E. Sakamoto T. Kondo K. (2014). Viral disruption of olfactory progenitors is exacerbated in allergic mice. *Int. Immunopharmacol.* 22 242–247. 10.1016/j.intimp.2014.06.034 24998164 PMC4129161

[B38] Ueha R. Shichino S. Ueha S. Kondo K. Kikuta S. Nishijima H. (2018a). Reduction of proliferating olfactory cells and low expression of extracellular matrix genes are hallmarks of the aged olfactory mucosa. *Front. Aging Neurosci.* 10:86. 10.3389/fnagi.2018.00086 29636678 PMC5880952

[B39] Ueha R. Ueha S. Kondo K. Kikuta S. Yamasoba T. (2018b). Cigarette smoke-induced cell death causes persistent olfactory dysfunction in aged mice. *Front. Aging Neurosci.* 10:183. 10.3389/fnagi.2018.00183 29950987 PMC6008309

[B40] Ueha R. Ueha S. Kondo K. Sakamoto T. Kikuta S. Kanaya K. (2016a). Damage to olfactory progenitor cells is involved in cigarette smoke-induced olfactory dysfunction in mice. *Am. J. Pathol.* 186 579–586. 10.1016/j.ajpath.2015.11.009 26806086

[B41] Ueha R. Ueha S. Sakamoto T. Kanaya K. Suzukawa K. Nishijima H. (2016b). Cigarette smoke delays regeneration of the olfactory epithelium in mice. *Neurotox. Res.* 30 213–224. 10.1007/s12640-016-9617-5 27003941

[B42] Wang Y. Bai C. Li K. Adler K. B. Wang X. (2008). Role of airway epithelial cells in development of asthma and allergic rhinitis. *Respir. Med.* 102 949–955. 10.1016/j.rmed.2008.01.017 18339528

[B43] Watanabe K. Fujimura M. Kasahara K. Yasui M. Myou S. Kita T. (2002). Acute eosinophilic pneumonia following cigarette smoking: a case report including cigarette-smoking challenge test. *Intern. Med.* 41 1016–1020. 10.2169/internalmedicine.41.1016 12487181

[B44] Wolfensberger M. Hummel T. (2002). Anti-inflammatory and surgical therapy of olfactory disorders related to sino-nasal disease. *Chem. Senses* 27 617–622. 10.1093/chemse/27.7.617 12200341

